# Association of abnormal electrocardiograph metrics with prolonged recovery time in incident hemodialysis patients

**DOI:** 10.1186/s12882-022-02664-3

**Published:** 2022-01-27

**Authors:** Jacqueline Watt, Jessica Fitzpatrick, Stephen M. Sozio, Bernard G. Jaar, Michelle M. Estrella, Larisa G. Tereshchenko, Jose M. Monroy-Trujillo, Michael Walsh, Rulan S. Parekh

**Affiliations:** 1 Child Health Evaluative Sciences, The Hospital for Sick Children, Toronto, Ontario Canada; 2 Department of Medicine, Johns Hopkins University School of Medicine, Baltimore, MD USA; 3 Welch Center for Prevention, Epidemiology, and Clinical Research, Baltimore, MD USA; 4 Department of Epidemiology, Bloomberg School of Public Health, Johns Hopkins University, Baltimore, MD USA; 5 Nephrology Center of Maryland, Baltimore, MD USA; 6 Kidney Health Research Collaborative, Department of Medicine, University of California, San Francisco, USA; 7 Department of Medicine, San Francisco VA Medical Center, San Francisco, CA USA; 8 Knight Cardiovascular Institute, Department of Medicine, Oregon Health and Science University, Portland, Oregon USA; 9 Division of Nephrology, Department of Medicine, McMaster University, Hamilton, Ontario Canada; 10 Population Health Research Institute, McMaster University, Hamilton, Ontario Canada; 11 Department of Health Research Methods, Evidence and Impact, McMaster University, Hamilton, Ontario Canada; 12 Division of Nephrology, Department of Pediatrics and Medicine, The Hospital for Sick Children, University Health Network and University of Toronto, 555 University Ave, Toronto, ON M5G 1X8 Canada

**Keywords:** Recovery time, Arrhythmias, Sudden cardiac death, Electrocardiography, QT interval, QTc, Dialysis, Incident, Hemodialysis, ESRD

## Abstract

**Background:**

Patients receiving intermittent hemodialysis have variable times of recovery to feeling better after dialysis. QT prolongation, a precursor to clinical and subclinical cardiovascular events, may contribute to delayed recovery time. We hypothesized that abnormal electrocardiographic parameters indicating perturbations in ventricular action are associated with longer recovery times thus impacting a patient-centered quality of life.

**Methods:**

Among 242 incident in-center hemodialysis participants from the Predictors of Arrhythmic and Cardiovascular Risk in End Stage Renal Disease (PACE) study, corrected QT interval (QTc), QRST angle and heart rate variance were measured on non-dialysis days using a standard 5-min electrocardiograph recording. Left ventricular hypertrophy (LVH) was defined using the Cornell voltage product. Recovery time was ascertained during a phone interview with a standardized validated questionnaire. Associations between QTc, QRST angle, heart rate variance, and LVH and natural log-transformed recovery time were examined using linear regression adjusted for participant characteristics and electrolytes.

**Results:**

Mean age was 55 (standard deviation 13) years, 55% were male, 72% were African American. Longer QTc interval was associated with increased recovery time (per 10 ms increase in QTc, recovery time increased by 6.2%; 95% confidence interval: 0.0–10.5). QRST angle, heart rate, heart rate variability and LVH were not significantly associated with recovery time.

**Conclusion:**

Longer QTc intervals are associated with longer recovery time independent of serum electrolytes. This supports a relationship between a patient’s underlying arrhythmic status and time to recovery after hemodialysis. Future studies will determine if maneuvers to reduce QTc improves recovery time and quality of life of patients on hemodialysis.

**Supplementary Information:**

The online version contains supplementary material available at 10.1186/s12882-022-02664-3.

## Background

Dialysis treatment has a major impact on patients’ quality of life [1]. Post-dialysis recovery time is associated with reduced quality of life and increased risk of mortality [2, 3]. Recovery time in the context of post-dialysis fatigue, defined as how long it takes to recover from a hemodialysis session, is a simple and effective assessment tool that is easily interpretable, stable over time, and sensitive to change [4]. Post-dialysis fatigue is an ill-defined term that is generalized into two parts: mental fatigue and physical fatigue [5]. Mental fatigue includes an inability to concentrate or follow along with a conversation, while physical fatigue includes a lack of energy and inability to complete daily tasks [5]. Both aspects are a detriment to a patient’s quality of life [6]; however, a clear physiological mechanism for these symptoms remains unknown.

Recovery time is a complex construct with several potential pathophysiological mechanisms that are not well understood. For example dialysis treatment, underlying comorbidities, serum electrolyte levels and volume of fluid removed during a hemodialysis session may contribute or exacerbate underlying cardiac and brain dysfunction [5, 7–9]. Additionally, low health-related quality of life scores have been associated with reduced heart rate variability and patient-reported recovery time [1–3, 10, 11]. End stage kidney disease patients receiving hemodialysis are also at high risk of another complex construct, sudden cardiac death, which is commonly related to a lethal arrythmia [7, 12, 13]. Monitoring of arrhythmias can be done using electrocardiograph (ECG) measurements, wherein a longer QT interval positively correlates with risk of fatal arrhythmias [14]. Although strong evidence exists to support an association between hemodialysis patients’ risk for arrhythmias and physiological measurements, there is limited information on the ability of these physiological measurements to predict patient-reported data, such as recovery time [2]. Studies examining chronic hemodialysis patients’ risk for clinically significant arrhythmias have shown a high incidence of arrhythmias occurring during and shortly after receiving the first hemodialysis session of the week as well as 12 h pre-hemodialysis [7, 12, 13, 15–17].

Arrhythmic potential may be a precursor to clinical and subclinical cardiovascular events, which may then contribute to delayed recovery time. We hypothesize that patients with increased arrhythmic potential experience delayed recovery times. The study objective was to assess the association of various ECG metrics, including corrected QT interval (QTc), QRST angle heart rate variance and, left ventricular hypertrophy with patient-reported recovery time within an incident in-center hemodialysis cohort.

## Methods

### Study design and population

This is a substudy of a longitudinal cohort of incident in-center hemodialysis participants from the Predictors of Arrhythmic and Cardiovascular Risk in End Stage Renal Disease (PACE) study. The study protocol was previously described in detail elsewhere [18]. Briefly, PACE was a prospective study of incident ESRD in-center hemodialysis participants enrolled from 27 outpatient dialysis units in the greater Baltimore area. Participants received regular outpatient thrice weekly hemodialysis. Inclusion criteria were: 1) ≥ 18 years of age and 2) ability to speak English. Exclusion criteria were: 1) institutionalized persons, 2) persons with a cancer diagnosis other than nonmelanoma skin cancer, 3) persons with a pacemaker or an automatic implantable cardioverter defibrillator, and 4) pregnant or nursing women. Participants who completed a baseline cardiovascular clinic visit and telephone interview were eligible for the present study. Participants were excluded from this substudy if they had detectable atrial fibrillation by ECG during the baseline study clinic visit, did not complete at least one ECG assessment, or did not complete a phone interview. Of the 571 incident hemodialysis participants enrolled in the PACE study, 365 (64%) participants completed a telephone interview. The study excluded individuals (*N* = 115) who were enrolled prior to inclusion of the telephone call in the study protocol. After additional exclusion of participants without ECG measurements (*N* = 26), the final sample included 242 participants.

The institutional review board of the Johns Hopkins School of Medicine, MedStar Health Systems, and the medical doctor of each dialysis unit approved the study protocol. All methods were carried out in accordance with relevant guidelines and regulations.

### Data collection

Participants completed standardized self-report questionnaires in the dialysis unit and at the Johns Hopkins Institute for Clinical and Translational Research (ICTR) clinic visit at baseline. Cardiac evaluations conducted on a non-dialysis day, including ECG, were performed by trained technologists or study staff at the ICTR baseline clinic visit. DaVita Clinical Research and MedStar Health Systems provided additional detailed hemodialysis treatment parameters and laboratory data.

### QT interval, QRST angle, heart rate variability and left ventricular hypertrophy

The ECG exposures of interest, corrected QT interval, spatial QRST angle were measured from 12-lead ECG. Heart rate variance was measured on 5-min ECG and was used to measure heart rate variability, the amount of variation in time between each heartbeat. All ECG recordings were performed on a non-dialysis day. QT interval was measured using signal-averaged ECG (Norav Medical LTD, Thornhill, ON, Canada) and corrected to QTc using Bazett’s formula [19]. Left ventricular hypertrophy (LVH) was defined as a Cornell voltage product of (S wave amplitude in V3 (SV3) + R wave in aVL (RaVL)) x QRS duration ≥244.0 mVms for males and (SV3 + (RaVL + 0.8 mV)) x QRS duration ≥244.0 mVms for females [20]. Spatial QRST angle was measured as previously described [21].

### Outcome

Post-dialysis recovery time, measured in minutes, was collected during a standardized telephone interview from participants’ answers to the question and using a validated question: “How long does it take you to recover from a dialysis session?” [4]. Recovery time collected during the telephone interview conducted closest to the participants’ first visit at the ICTR was used in this analysis. Median time between dialysis initiation and study enrollment was 3.5 months (interquartile range [IQR]: 2.7, 5.0), median time between study enrollment and assessment of post-dialysis recovery time was 6.6 months (IQR: 5.7, 8.5), and median time between dialysis initiation and recovery time assessment was 10.8 months (IQR: 9.2, 13.7).

### Additional participant characteristics

Age (continuous), sex, self-reported race (African American or Non-African American), depression severity and comorbidities derived from a combination of self-report questionnaires and detailed chart review of medical records were collected at baseline. Depression severity was measured as the total score of the Patient Health Questionaire-9 and used as a continuous variable in analysis [22]. Comorbidities assessed as present or absent included hypertension, hypercholesterolemia, diabetes, coronary artery disease, and congestive heart failure. Comorbidities were adjudicated by the PACE endpoint committee and used to calculate the Charlson comorbidity index.

Use of antihypertensive medications were recorded during study visits. Serum ionized calcium and magnesium levels (continuous variables) were analyzed from blood samples collected at study baseline on a non-dialysis day. Serum potassium levels were calculated as a 90-day average from study baseline. Seated systolic and diastolic blood pressure were collected on a non-dialysis day and averaged over 3 readings from different days. Baseline left ventricular mass was estimated by ECG and left ventricular mass index (LVMI; left ventricular mass/body surface area) was calculated using Devereux’s formula [23].

### Statistical analysis

All continuous variables with normal distributions were described using means (standard deviation [SD]) and non-normal distributions using median (IQR). Categorical variables were examined using frequencies (%).

Associations of QT interval, QRST angle, heart rate variability and LVH with recovery time were examined using linear regression. Log transformation of the recovery time plus 1 (i.e. natural log (recovery time + 1)) was performed to normalize distribution for a linear regression analyses. Associations between each exposure and recovery time were examined separately using univariable and multivariable linear regression. Potential variables for the multivariable models were selected based on previous literature describing association with long QT, heart rate variability and recovery time. Final variables included in the multivariable model were determined based on changes in effect-size from a forward selection model which included age, sex, race, congestive heart failure, CRP, diabetes, total depression score, LVMI, Charlson comorbidity index, serum ionized calcium, serum magnesium, and the use of renin-angiotensin-aldosterone system blockades, beta-blockers, diuretics, or antihypertensive medication. The final model includes age, sex, race, total depression score, LVMI, Charlson comorbidity index, serum ionized calcium, serum magnesium, and the use of antihypertensive medication.

Interactions between the exposure variables with sex (male vs. female), age (≤55 vs. > 55), and LVH were tested based on findings from previous studies [2, 5, 14, 24]. Interactions with race (African American vs. non-African American) were also tested due to a high proportion of African Americans in the PACE population. Sensitivity analysis was performed using QTc defined using Fridericia’s formula to account for higher heart rates [25]. To test the robustness of our results, the analysis was repeated with recovery time dichotomized at the sample median (median = 20 min) using logistic regression as well as with recovery time restricted to being collected within six months of cardiac evaluation. The Hosmer and Lemeshow’s goodness-of-fit test and residual versus fitted plots were used to assess model fit and heteroskedasticity, respectively. All missing covariates were imputed using the multiple imputation by chained equations method with 20 imputations and 20 iterations [23]. The imputed variables were total depression score (5%), serum ionized calcium (8%), serum magnesium (7%), and antihypertensive medication use (9%). R^2^ values for the final adjusted models were calculated and averaged across 20 imputations (QT interval, R^2^ = 7.5%; QTc interval, R^2^ = 8.7%; QRST angle, R^2^ = 5.7%; Heart rate, R^2^ = 5.8%; Heart rate variance, R^2^ = 7.0%; Left ventricular hypertrophy, R^2^ = 5.7%).

A two-tailed *P* value of < 0.05 was considered significant for all analyses and were performed using STATA 16.0 (College Station, Texas).

## Results

### Participant characteristics

The incident cohort had a mean age of 55 (SD 13) years, 133 (55%) were male, 175 (72%) were African American, the median total depression score was 4 (IQR: 1, 8), 138 (57%) had diabetes, and 97 (40%) had congestive heart failure. Participants’ ECG values did not materially differ between their baseline and first follow-up visit. The mean QT interval was 452.5 (48.4) ms, and the median heart rate variance was 380.3 ms^2^ (IQR: 128.8, 1228.9) (Table 1).

**Table 1:** Baseline characteristics of the PACE study cohort (2009–2012)

Characteristics Mean (SD) or % or Median (IQR)	Normal QTc (*N* = 40)	Prolonged QTc^a^ (*N* = 202)	Overall (*N* = 242)
**Demographics**			
Age (years)	54 (16)	55 (13)	55 (13)
Male	16 (40%)	117 (58%)	133 (55%)
African American	29 (73)	146 (72%)	175 (72%)
PHQ-9 Total Depression Score	4 (1, 7)	4 (1, 8)	4 (1, 8)
**ECG Metrics**			
QT Interval (ms)	415.0 (29.3)	459.9 (48.0)	452.5 (48.4)
QTc (ms)	433.8 (19.5)	498.9 (43.1)	488.1 (46.9)
QRST Angle (degrees)	7.2 (5.2)	9.3 (4.4)	9.0 (4.6)
Heart Rate (ms)	92.2 (13.5)	85.7 (13.4)	86.8 (13.6)
Heart Rate Variance (ms^2^)	350.9 (107.4, 1103.8)	640.5 (202.7, 1840.5)	380.3 (128.8, 1228.9)
LVH^c^	5 (12.8%)	27 (14.8%)	32 (14.5%)
**Cardiovascular Risk Factors**			
LVMI^d^ (g/m^2^)	134.7 (52.6)	140.5 (45.0)	139.5 (46.3)
CRP (μg/mL)	3.2 (1.6, 7.4)	6.4 (2.6, 15.0)	5.8 (2.4, 14.9)
Diabetes	17 (43%)	121 (60%)	138 (57%)
RAAS^b^	17 (46%)	81 (44%)	98 (44%)
Beta Blockers	21 (53%)	131 (65%)	152 (63%)
Alpha-Blocker, Calcium Channel Blocker, Vasodilator	31 (84%)	142 (77%)	173 (78%)
Diuretic	4 (11%)	46 (23%)	50 (23%)
Congestive Heart Failure	15 (38%)	82 (41%)	97 (40%)
Charlson Comorbidity Index	3 (2, 5)	4 (3, 6)	4 (3, 6)
Baseline Length of Hemodialysis Treatment	215.5 (24.0)	215.9 (19.7)	215.9 (20.3)
Interdialytic Weight Gain	1.8 (0.6)	2.2 (0.9)	2.1 (0.9)
Use of QT Prolonging Medications^e^	11 (28%)	74 (37%)	85 (35%)
Non-dialysis Seated Systolic Blood Pressure	129.9 (20.7)	137.6 (25.6)	136.4 (25.0)
Non-dialysis Seated Diastolic Blood Pressure	69.4 (14.0)	74.6 (13.7)	73.7 (13.8)
**Outcome**			
Recovery Time (min)	10 (7.5, 30)	30 (10, 120)	20 (10, 60)

^a^ Defined as > 440 ms for males and > 460 ms for females [26]

^b^ Renin-angiotensin-aldosterone system blockades

^c^ Left ventricular hypertrophy defined using the Cornell voltage product [20]

^d^ Left ventricular mass index

^e^ QT prolonging medications include furosemide, ritonavir, sertraline, trazodone, escitalopram, tramadol, esomeprazole, pantoprazole, lansoprazole, and metoclopramide

### Outcome

The median recovery time was 20 (IQR: 10, 60) minutes.

### QT interval, QRST angle, heart rate variability, left ventricular hypertrophy and recovery time

**Fig. 1:**
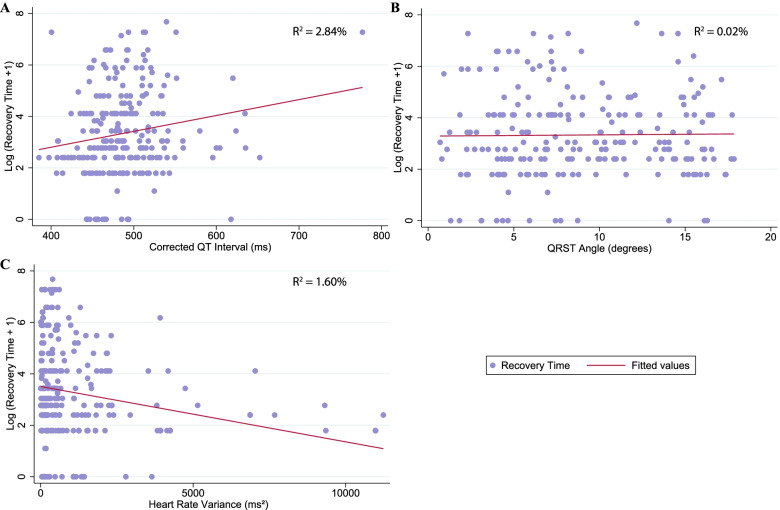
ECG measurements over recovery time in minutes, model includes recovery time and one of the main exposures. **A** The distribution of QTc interval lengths. **B** The distribution of QRST angles. **C** The distribution of heart rate variance

**Fig. 2:**
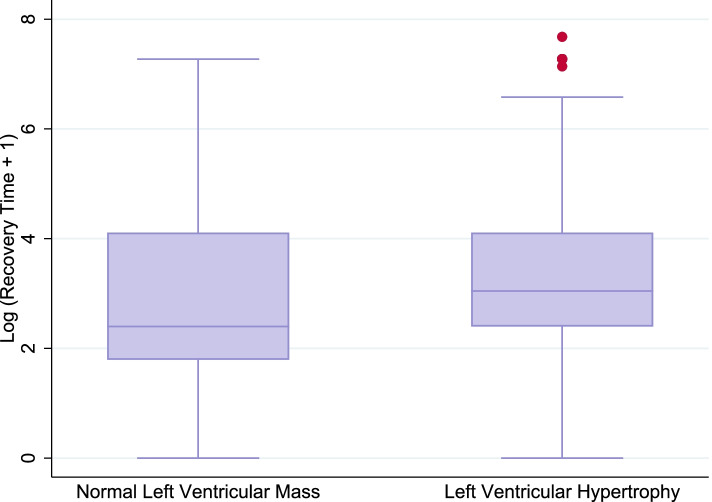
Recovery time over left ventricular hypertrophy. Left ventricular hypertrophy is defined using the Cornell voltage product [20]

In univariable analyses (Figs. 1 and 2), longer QTc and heart rate variability were significantly associated with recovery time (Table 2). QT and QTc remained significantly associated with longer recovery time after adjusting for demographic characteristics, comorbidity, depression severity, antihypertensive medication use, LVMI, serum ionized calcium, and serum magnesium (per 10.0 ms longer QT and QTc; difference = 5.1, 95% confidence interval [CI]: 0.0, 10.5; 6.2, 95% CI: 2.0, 11.6, respectively). There were no significant independent associations between QRST angle, heart rate, heart rate variability and LVH with recovery time after multivariable analysis (Table 2; difference = 0.0, 95% CI: − 4.9, 5.1; − 4.9, 95% CI: − 19.7, 12.7; − 1.0, 95% CI: − 2.0, 0.0; 10.5, 95% CI: − 44.0, 118.1, respectively).

**Table 2:** Association of ECG metrics with recovery time (RT) after dialysis

Exposure	Model 1				Model 2				Model 3			
	N	RT Difference	95% CI	P	N	RT Difference	95% CI	P	N	RT Difference	95% CI	P
**QT Interval**, per 10.0 ms increase	242	4.1	(−1.0, 8.3)	0.1	242	5.1	(−1.0, 9.4)	0.06	**242**	**5.1**	**(0.0, 10.5)**	**0.04**
**QTc Interval**, per 10.0 ms increase	242	6.2	(2.0, 11.6)	0.009	242	6.2	(1.0, 11.6)	0.01	**242**	**6.2**	**(2.0, 11.6)**	**0.007**
**QRST angle**, per 10 degree increase	221	1.0	(−3.9, 5.1)	0.9	221	1.0	(−3.9, 6.2)	0.8	221	0.0	(−4.9, 5.1)	0.9
**Heart rate**, per 100 ms increase	242	−7.7	(−21.3, 8.3)	0.3	242	−4.9	(−19.7, 17.4)	0.5	242	−4.9	(− 19.7, 12.7)	0.6
**Heart Rate Variance**, per 100 ms^2^ increase	242	−1.0	(−3.0, 0.0)	0.05	242	−1.0	(−3.0, 0.0)	0.05	242	−1.0	(−2.0, 0.0)	0.08
**Left Ventricular Hypertrophy**^a^	242	12.7	(−40.0, 113.8)	0.7	242	16.2	(−38.7, 120.3)	0.6	242	10.5	(−44.0, 118.1)	0.8

Model 1 includes the main exposure (one of the ECG measurements)

Model 2 includes model 1, age, sex, and race

Model 3 includes model 2, total depression score, LVMI, Charlson comorbidity index, serum ionized calcium, serum magnesium, and the use of antihypertensive medication

^a^For left ventricular hypertrophy, Model 3 does not include LVMI

### Subgroup analyses

Association between QTc and recovery time did not differ by sex (P_interaction_ = 0.88), age (P_interaction_ = 0.23), or race (P_interaction_ = 0.52; Supplementary Table 1 and 2). Similarly, there was no evidence of effect modification of QT interval, QRST angle, heart rate, heart rate variability or LVH and recovery time (all P_interaction_ > 0.1) by age, sex, or race. Additionally, there was no evidence of effect modification of QT interval, QRST angle, heart rate, heart rate variability by LVH (all P_interaction_ > 0.3).

### Sensitivity analyses

Dichotomizing recovery time at the sample median did not change inferences on the association between QTc, QRST angle, heart rate variability or LVH and recovery time (Supplementary Table 3). Additionally, restricting recovery time to those reported within six months of the participants’ first visit at the ICTR did not change the associations between all exposures and recovery time (Supplementary Table 4). The association of QT interval and recovery time was also re-examined using Fridericia’s formula to correct the QT interval (Supplementary Table 5) and similar results were obtained.

We note there is one outlier with severe QT prolongation > 700 ms. After removing the outlier and repeating the analysis, QTc remained significantly associated with longer recovery time with similar estimates (Supplementary Table 6). The association of QTc, QRST angle, heart rate variability or LVH and dialysis recovery time were also similar when separately adjusted for baseline length of hemodialysis treatment, relative fluid removal, use of QT prolonging medications, and serum potassium levels (Supplementary Table 7).

## Discussion

Quality of life for dialysis patients especially after dialysis is an important metric of resolution of symptoms and associated with long-term outcomes [1–3, 6]. Patients on hemodialysis with longer QTc had longer recovery times. QTc is a potentially modifiable factor which may ameliorate recovery time and improve quality of life for patients receiving hemodialysis. Recovery time after dialysis is an important patient-centered outcome that is readily obtained and easy for patients to understand. The association of prolonged QTc and recovery time persisted even after adjustment of several important clinical confounders. None of the other ECG metrics were associated with recovery time.

QTc prolongation is defined as > 440 ms in males and > 460 ms in females and are typically affected by electrolyte changes [26]. In our population, the mean normal QTc for males was 420.6 ms (16.5) and 442.6 ms (16.3) for females prior to dialysis initiation. Based on the study findings, recovery time increases by 6% per 10 ms increase in QTc. On average, participants recovered with a median of 20 (IQR: 10, 60) minutes and if recovery time increase of 14 min could potentially indicate a prolonged QTc interval and thus increased risk for lethal cardiovascular events.

Recovery time associates with an increased dialysis stress score, increased risk of hospitalization and lower survival [11]. It is also known that osmotic shifts between extra- and intracellular fluid compartments and fluctuating electrolyte concentrations contribute to the stress of dialysis [2]. These fluctuating electrolyte concentrations, specifically those of calcium and potassium, are important in ventricular conduction and prolong the QTc interval [7]. Given recent advances in understanding how frequent arrhythmias are during and up to 72 h after hemodialysis [15], it is not unreasonable to postulate that the occurrence of post-dialysis arrhythmias occurs more commonly in patients with longer QTc and that the resulting symptoms may be perceived by patients as contributing to their recovery time [7, 27]. It is important to recognize that this association does not imply causation, as either QTc or recovery time prolongation could reflect various underlying mechanism, potentially related to increased dialysis stress with change in physiology during dialysis.

The relationship between lower health-related quality of life, risk of mortality and recovery time is well-established [1–3, 24]. However, the pathophysiology and clinical factors associated with recovery time are not completely understood. Longer recovery times were associated with female sex, older age, dialysis vintage, higher body mass index, and unemployment status [2, 28]. Greater self-reported depression scores and low dialysate potassium were also associated with extended recovery times [29, 30]. Conversely, similar studies have found no associations with recovery time, sex, age, and number of comorbidities [29, 31]. We found no association with age, however the effect of QTc on recovery time is not present in participants ≤55 years (Supplementary Table 2). This could be related to an increased prevalence of frailty in older individuals [32]. Therefore, a patient’s ability to cope with the physiological stress of dialysis may be a factor in how long it takes to recover after hemodialysis.

Based on our results, we should now consider adding prolonged QTc interval as a risk factor for delayed recovery after dialysis. If altering the hemodialysis treatment, with changes to electrolytes, or medications in ways that predictably reduce QTc (and therefore reduce the risk of lethal arrhythmias) may reduce recovery time, it could be of significant clinical importance to monitor a patient’s recovery time as a method of improving their cardiac health.

Limitations of this study include the time difference between when recovery time was reported and when ECG measurements were obtained. ECG parameters on non-dialysis days are generally stable over one year [33]; therefore, we do not expect the time lag between exposure and outcome assessments to materially affect the results. ECG assessments were also limited to interdialytic measures. Additionally, due to recovery time being patient reported, there is subjectivity inherent to the patients’ answers, however, this is validated patient reported measure [4]. Finally, although we had adequate statistical power to detect independent associations, we may not have had sufficient power to detect interactions. Despite these limitations, there are many important strengths of this study that should be acknowledged. This was a large prospective cohort study of incident in-center hemodialysis patients, including a large minority under-studied population, with standardized cardiovascular and clinical measures. Compared to the United States Renal Data Systems, PACE participants are younger and a larger portion are Black and/or African American [18].

## Conclusions

Prolonged QTc is associated with longer recovery times among adults initiating hemodialysis independent of electrolytes. These findings support an association between arrhythmic potential and symptoms of recovery after hemodialysis. Specifically, intra and post-dialytic arrhythmias may contribute to feeling unwell after dialysis. Future studies will need to determine if increasing maneuvers to reduce QTc improves recovery time and overall quality of life of patients on hemodialysis.

## Supplementary Information

**Additional file 1: Supplementary Table 1**: Association of post-dialysis recovery time (RT) with ECG measurements QT interval, QTc interval, and heart rate variability, by sex (male vs. female).

**Additional file 2: Supplementary Table 2**: Association of post-dialysis recovery time (RT) with ECG measurements QT interval, QTc interval, and heart rate variability, by age (≤55 years old vs. > 55 years old).

**Additional file 3: Supplementary Table 3**: Association of ECG measurements with post-dialysis recovery time dichotomized at the median (median = 20).

**Additional file 4: Supplementary Table 4**: Association of ECG measurements with post-dialysis recovery time (RT) recorded within six months (±180 days).

**Additional file 5: Supplementary Table 5**: Association of post-dialysis recovery time (RT) with QTc using Fridericia’s formula.

**Additional file 6: Supplementary Table 6**: Association of ECG measurements without outlier.

**Additional file 7: Supplementary Table 7**: Association of ECG measurements with recovery time adjusted for baseline length of hemodialysis treatment, relative fluid removal, use of QT prolonging medications*, and serum potassium levels.

## Data Availability

The datasets used and/or analysed during the current study are available from the corresponding author on reasonable request.

## Abbreviations

ECG: Electrocardiograph
PACE: Predictors of Arrhythmic and Cardiovascular Risk in End Stage Renal Disease
ICTR: Institute for Clinical and Translational Research
QTc: Corrected QT interval
LVH: Left ventricular hypertrophy
IQR: Interquartile range
LVMI: Left ventricular mass index
SD: Standard deviation
