# Supplementary material for: Association of abnormal electrocardiograph metrics with prolonged recovery time in incident hemodialysis patients
Source: BMC Nephrol. 2022 Jan 27;23:46. doi: 10.1186/s12882-022-02664-3 (PMC8796483; doi:10.1186/s12882-022-02664-3)
Supplement: Supplementary file 1 — Additional file 1: Supplementary Table 1: Association of post-dialysis recovery time (RT) with ECG measurements QT interval, QTc interval, and heart rate variability, by sex (male vs. female). [file 12882_2022_2664_MOESM1_ESM.docx]

| **Exposure** | **Males** | | | | **Females** | | | | |
| --- | --- | --- | --- | --- | --- | --- | --- | --- | --- |
|  | N | RT Difference | 95% CI | P | N | RT Difference | 95% CI | P | |
| **QT Interval**, per 10.0 ms increase | 133 | 4.8 | (-1.3, 11.2) | 0.1 | 109 | 6.0 | (-3.4, 16.3) | 0.2 | |
| **QTc Interval**, per 10.0 ms increase | **133** | **7.6** | **(1.9, 13.6)** | **0.01** | 109 | 4.6 | (-5.0, 15.1) | 0.4 | |
| **QRST angle**, per 10 degree increase | 124 | -0.4 | (-7.0, 6.7) | 0.9 | 97 | 1.7 | (-6.4, 10.4) | 0.7 | |
| **Heart rate**, per 100 ms increase | 133 | -11.5 | (-30.0, 11.9) | 0.3 | 109 | 10.0 | (-19.2, 49.6) | 0.5 | |
| **Heart Rate Variance**, per 100 ms^2^ increase | **133** | **-1.4** | **(-2.6, -0.3)** | **0.02** | 109 | -0.6 | (-1.8, 0.6) | 0.3 | |
| **Left Ventricular Hypertrophy^†^** | 133 | 24.3 | (-47.9, 196.7) | 0.6 | 109 | 41.7 | (-44.7, 263.3) | 0.4 | |
| Models include the main exposure (one of the ECG measurements), age, sex, race, total depression score, LVMI, Charlson comorbidity index, serum ionized calcium, serum magnesium, and the use of antihypertensive medication  **^†^**For left ventricular hypertrophy, Model 3 does not include LVMI | | | | | | | | |  |
