# Supplementary material for: Association of abnormal electrocardiograph metrics with prolonged recovery time in incident hemodialysis patients
Source: BMC Nephrol. 2022 Jan 27;23:46. doi: 10.1186/s12882-022-02664-3 (PMC8796483; doi:10.1186/s12882-022-02664-3)
Supplement: Supplementary file 3 — Additional file 3: Supplementary Table 3: Association of ECG measurements with post-dialysis recovery time dichotomized at the median (median = 20). [file 12882_2022_2664_MOESM3_ESM.docx]

| **Exposure** | **Model 1** | | | | **Model 2** | | | | **Model 3** | | | |
| --- | --- | --- | --- | --- | --- | --- | --- | --- | --- | --- | --- | --- |
|  | N | OR | 95% CI | P | N | OR | 95% CI | P | N | OR | 95% CI | P |
| **QT Interval**, per 10.0 ms increase | 242 | 1.02 | (0.96, 1.07) | 0.6 | 242 | 1.04 | (0.98, 1.10) | 0.2 | 242 | 1.04 | (0.98, 1.10) | 0.2 |
| **QTc Interval**, per 10.0 ms increase | 242 | 1.05 | (0.99, 1.11) | 0.1 | 242 | 1.05 | (0.99, 1.11) | 0.1 | 242 | 1.06 | (1.00, 1.13) | 0.06 |
| **QRST angle**, per 10 degree increase | 221 | 1.00 | (0.94, 1.06) | 0.9 | 221 | 1.01 | (0.95, 1.07) | 0.7 | 221 | 1.00 | (0.94, 1.07) | 0.9 |
| **Heart rate**, per 100 ms increase | 242 | 0.89 | (0.74, 1.08) | 0.2 | 242 | 0.96 | (0.79, 1.17) | 0.7 | 242 | 0.94 | (0.77, 1.16) | 0.6 |
| **Heart Rate Variance**, per 100 ms^2^ increase | 242 | 0.98 | (0.96, 0.99) | 0.01 | 242 | 0.98 | (0.96, 0.99) | 0.01 | **242** | **0.97** | **(0.95, 0.99)** | **0.01** |
| **Left Ventricular Hypertrophy^†^** | 242 | 1.61 | (0.75, 3.48) | 0.2 | 242 | 1.64 | (0.74, 3.61) | 0.2 | 242 | 1.54 | (0.65, 3.63) | 0.3 |
| Model 1 includes the main exposure (one of the ECG measurements)  Model 2 includes model 1, age, sex, and race  Model 3 includes model 2, total depression score, LVMI, Charlson comorbidity index, serum ionized calcium, serum magnesium, and the use of antihypertensive medication  **^†^**For left ventricular hypertrophy, Model 3 does not include LVMI | | | | | | | | | | | | |
