# Supplementary material for: Association of abnormal electrocardiograph metrics with prolonged recovery time in incident hemodialysis patients
Source: BMC Nephrol. 2022 Jan 27;23:46. doi: 10.1186/s12882-022-02664-3 (PMC8796483; doi:10.1186/s12882-022-02664-3)
Supplement: Supplementary file 4 — Additional file 4: Supplementary Table 4: Association of ECG measurements with post-dialysis recovery time (RT) recorded within six months (±180 days). [file 12882_2022_2664_MOESM4_ESM.docx]

| **Exposure** | **Model 1** | | | | **Model 2** | | | | **Model 3** | | | | |
| --- | --- | --- | --- | --- | --- | --- | --- | --- | --- | --- | --- | --- | --- |
|  | N | RT Difference | 95% CI | P | N | RT Difference | 95% CI | P | N | RT Difference | 95% CI | P | |
| **QT Interval**, per 10.0 ms increase | 71 | 11.6 | (3.0, 20.9) | 0.01 | 71 | 10.5 | (2.0, 20.9) | 0.02 | **71** | **10.5** | **(0.0, 20.9)** | **0.05** | |
| **QTc Interval**, per 10.0 ms increase | 71 | 12.7 | (3.0, 23.4) | 0.01 | 71 | 12.7 | (3.0, 22.1) | 0.01 | **71** | **1.0** | **(11.6, 22.1)** | **0.03** | |
| **QRST angle**, per 10 degree increase | 65 | 0.0 | (-10.4, 10.5) | 0.9 | 65 | -1.0 | (-11.3, 9.4) | 0.8 | 65 | 1.0 | (1.0, 12.7) | 0.8 | |
| **Heart rate**, per 100 ms increase | 71 | 6.2 | (-25.2, 50.7) | 0.8 | 71 | 6.2 | (-29.5, 46.2) | 0.9 | 71 | -5.8 | (-36.2, 40.5) | 0.8 | |
| **Heart Rate Variance**, per 100 ms^2^ increase | 71 | -2.0 | (-4.9, 1.0) | 0.2 | 71 | -2.0 | (-4.9, 1.0) | 0.2 | 71 | -2.0 | (-4.9, 1.0) | 0.2 | |
| **Left Ventricular Hypertrophy^†^** | 71 | 99.4 | (-26.7, 436.6) | 0.5 | 71 | 75.1 | (-36.2, 380.7) | 0.5 | 71 | 153.5 | (-31.6, 839.3) | 0.3 | |
| Model 1 includes the main exposure (one of the ECG measurements)  Model 2 includes model 1, age, sex, and race  Model 3 includes model 2, total depression score, LVMI, Charlson comorbidity index, serum ionized calcium, serum magnesium, and the use of antihypertensive medication  **^†^**For left ventricular hypertrophy, Model 3 does not include LVMI | | | | | | | | | | | | |  |
