# Supplementary material for: Association of abnormal electrocardiograph metrics with prolonged recovery time in incident hemodialysis patients
Source: BMC Nephrol. 2022 Jan 27;23:46. doi: 10.1186/s12882-022-02664-3 (PMC8796483; doi:10.1186/s12882-022-02664-3)
Supplement: Supplementary file 5 — Additional file 5: Supplementary Table 5: Association of post-dialysis recovery time (RT) with QTc using Fridericia’s formula. [file 12882_2022_2664_MOESM5_ESM.docx]

| **Exposure** | **Model 1** | | | | **Model 2** | | | | **Model 3** | | | | |
| --- | --- | --- | --- | --- | --- | --- | --- | --- | --- | --- | --- | --- | --- |
|  | N | RT Difference | 95% CI | P | N | RT Difference | 95% CI | P | N | RT Difference | 95% CI | P | |
| **QTc Interval**, per 10.0 ms increase | 242 | 6.2 | (1.0, 11.6) | 0.02 | 242 | 6.5 | (1.0, 11.6) | 0.01 | **242** | **7.1** | **(2.0, 12.7)** | **0.01** | |
| Model 1 includes the main exposure (one of the ECG measurements)  Model 2 includes model 1, age, sex, and race  Model 3 includes model 2, total depression score, LVMI, Charlson comorbidity index, serum ionized calcium, serum magnesium, and the use of antihypertensive medication | | | | | | | | | | | | |  |
