# Supplementary material for: Association of abnormal electrocardiograph metrics with prolonged recovery time in incident hemodialysis patients
Source: BMC Nephrol. 2022 Jan 27;23:46. doi: 10.1186/s12882-022-02664-3 (PMC8796483; doi:10.1186/s12882-022-02664-3)
Supplement: Supplementary file 6 — Additional file 6: Supplementary Table 6: Association of ECG measurements without outlier. [file 12882_2022_2664_MOESM6_ESM.docx]

| **Exposure** | **Model 1** | | | | **Model 2** | | | | **Model 3** | | | |
| --- | --- | --- | --- | --- | --- | --- | --- | --- | --- | --- | --- | --- |
|  | N | RT Difference | 95% CI | P | N | RT Difference | 95% CI | P | N | RT Difference | 95% CI | P |
| **QT Interval**, per 10.0 ms increase | 241 | 1.9 | (-2.9, 6.9) | 0.4 | 241 | 3.0 | (-2.0, 8.2) | 0.3 | 241 | 3.7 | (-1.4, 9.2) | 0.2 |
| **QTc Interval**, per 10.0 ms increase | 241 | 4.9 | (-0.2, 10.3) | 0.06 | 241 | 4.8 | (-0.3, 10.2) | 0.07 | **241** | **5.4** | **(0.2, 11.0)** | **0.04** |
| **QRST angle**, per 10 degree increase | 220 | 0.7 | (-4.0, 5.7) | 0.8 | 220 | 0.9 | (-3.8, 5.9) | 0.7 | 220 | 0.6 | (-4.3, 5.7) | 0.8 |
| **Heart rate**, per 100 ms increase | 241 | -7.9 | (-21.4, 7.9) | 0.3 | 241 | -5.1 | (-19.7, 12.2) | 0.5 | 241 | -4.4 | (-19.3, 13.3) | 0.6 |
| **Heart Rate Variance**, per 100 ms^2^ increase | 241 | -1.2 | (-2.5, 0.0) | 0.05 | 241 | -1.2 | (-2.5, 0.0) | 0.05 | 241 | -1.2 | (-2.4, 0.1) | 0.08 |
| **Left Ventricular Hypertrophy^†^** | 241 | 18.9 | (-26.3, 91.6) | 0.5 | 241 | 17.6 | (-27.3, 90.2) | 0.5 | 241 | 3.0 | (-36.7, 67.7) | 0.9 |
| Model 1 includes the main exposure (one of the ECG measurements)  Model 2 includes model 1, age, sex, and race  Model 3 includes model 2, total depression score, LVMI, Charlson comorbidity index, serum ionized calcium, serum magnesium, and the use of antihypertensive medication  **^†^**For left ventricular hypertrophy, Model 3 does not include LVMI | | | | | | | | | | | | |
