# Supplementary material for: Association of abnormal electrocardiograph metrics with prolonged recovery time in incident hemodialysis patients
Source: BMC Nephrol. 2022 Jan 27;23:46. doi: 10.1186/s12882-022-02664-3 (PMC8796483; doi:10.1186/s12882-022-02664-3)
Supplement: Supplementary file 7 — Additional file 7: Supplementary Table 7: Association of ECG measurements with recovery time adjusted for baseline length of hemodialysis treatment, relative fluid removal, use of QT prolonging medications*, and serum potassium levels. [file 12882_2022_2664_MOESM7_ESM.docx]

| **Exposure** | **Model** | | | |
| --- | --- | --- | --- | --- |
|  | N | RT Difference | 95% CI | P |
| **QT Interval**, per 10.0 ms increase | **242** | **5.1** | **(0.3, 10.2)** | **0.04** |
| **QTc Interval**, per 10.0 ms increase | **242** | **6.7** | **(1.8, 11.9)** | **0.007** |
| **QRST angle**, per 10 degree increase | 221 | 0.2 | (-4.7, 5.4) | 0.9 |
| **Heart rate**, per 100 ms increase | 242 | -0.5 | (-2.2, 1.2) | 0.6 |
| **Heart Rate Variance**, per 100 ms^2^ increase | 242 | -1.1 | (-2.4, 0.2) | 0.09 |
| **Left Ventricular Hypertrophy^†^** | 242 | 7.0 | (-34.5, 74.6) | 0.8 |
| Model includes main exposure (one of the ECG measurements), age, sex, race, total depression score, LVMI, Charlson comorbidity index, serum ionized calcium, serum magnesium, use of antihypertensive medication, baseline length of hemodialysis treatment, relative fluid removal, and the use of QT prolonging medications  *QT prolonging medications include furosemide, ritonavir, sertraline, trazodone, escitalopram, tramadol, esomeprazole, pantoprazole, lansoprazole, and metoclopramide  **^†^**For left ventricular hypertrophy, Model does not include LVMI | | | | |
